# Investigating the attitude of psychiatrists towards the presence of stigma about COVID-19

**DOI:** 10.3389/fpsyt.2025.1553197

**Published:** 2025-04-02

**Authors:** Farhoud Moradi-Gorabpasi, Mojgan Khademi, Arsia Taghva, Shirin Shakeri, Leili Panaghi

**Affiliations:** ^1^ Shahid Beheshti University of Medical Sciences, Tehran, Iran; ^2^ Department of Child and Adolescent Psychiatry, Shahid Beheshti University of Medical Sciences, Tehran, Iran; ^3^ Department of Psychiatry, Faculty of Medicine, AJA University of Medical Sciences, Tehran, Iran; ^4^ Department of Community Medicine, Family Research Institute, Shahid Beheshti University, Tehran, Iran

**Keywords:** COVID-19, stigma, mental health, stigma questionnaire, psychiatrists

## Abstract

**Introduction:**

The COVID-19 pandemic has led to significant global disruption, resulting in increased stigma and discrimination towards certain communities and populations affected by the virus. Given that psychiatrists play a crucial role in both preventing and treating the complications associated with disease-related stigma, this study aims to examine their attitude towards the stigma associated with COVID-19.

**Methods:**

This research utilized a cross-sectional survey design to evaluate psychiatrists' attitudes towards COVID-19 stigma. We used a 15-item researcher-made questionnaire with scores ranging from 15 to 75. The questionnaire was distributed to 131 psychiatrists in Tehran (capital city of Iran) from April 9, 2023 to May 26, 2023, with responses collected voluntarily. Data were analyzed with descriptive statistics, independent t-tests, and one-way ANOVA to evaluate stigma attitudes across demographic variables using SPSS software (version 25).

**Results:**

The Cronbach's alpha for the COVID-19 Stigma Attitude Scale for Psychiatrists (CSASP) was determined to be 0.861, indicating strong reliability. Moreover, all questions achieved acceptable corrected item-total correlation values above 0.2. It was revealed that the highest and lowest recorded scores were 68 and 25, respectively (average 51.16 ± 8.83). Also 19 individuals (14.5%) exhibited a weak attitude, 41 individuals (31.3%) displayed a rather weak attitude, 54 individuals (41.2%) showed a rather strong attitude, and 17 individuals (13%) demonstrated a strong attitude toward the presence of COVID-19 stigma. Furthermore, 96 individuals (73.3%) recognized stigma surrounding COVID-19 at the onset of the pandemic; of these, 11 (11.5%) disagreed with the idea of current stigma, while 18 (18.7%) had no opinion. Thus, 67 participants (69.8%) still believe such a stigma exists. Finally, 83 respondents (63.3%) acknowledged stigma related to AIDS and leprosy, with 53 (63.8%) of them also believing in the stigma surrounding COVID-19.

**Conclusion:**

The findings highlighted various attitudes towards the ongoing stigma associated with COVID-19, with most psychiatrists recognizing its persistence throughout the pandemic. The research also points to the interconnectedness of stigma across different diseases, emphasizing common societal factors like fear, misinformation, and cultural biases. This underscores the crucial role psychiatrists play in addressing stigma and its effects on society.

## 1 Introduction

At the end of 2019, a new infectious disease, known as Coronavirus Disease 2019 (COVID-19), emerged as the most significant public health concern (1). On March 11, 2020, the World Health Organization (WHO) officially classified COVID-19 as a global pandemic. The pathogen demonstrated a rapid and extensive transmission, impacting millions globally. The estimated case fatality rate during this period was approximately 5.7%, highlighting the severe public health implications of the outbreak (2). As there is no approved vaccine or treatment for COVID-19, efforts to combat the disease have concentrated on preventing its spread. These efforts involved political decisions to implement social distancing measures together with public health education aimed at raising individual awareness about the disease and how to protect oneself (3). A survey of the general public in China examined the psychological effects of the COVID-19 outbreak's early phase. The results showed that 53.8% of the respondents experienced a moderate to severe psychological impact, 16.5% reported moderate to severe depressive symptoms, 28.8% faced moderate to severe anxiety, and 8.1% experienced moderate to severe stress. These mental health burdens were not only caused by the virus itself, but were also worsened by fears related to stigma, including social rejection, discrimination, and the belief that those infected were responsible for spreading the disease (4). The term "stigma" originally refers to a mark used to identify Greek slaves and distinguish them from free individuals. Over time, this association led "stigma" to signify a characteristic that is deeply discrediting. Interestingly, in Persian, the word "stigma" denotes a symbol inscribed on commercial documents (5). Stigma is a phenomenon that involves labeling, stereotyping, isolation, loss of status, and discrimination. Asymmetric relationships can worsen these components (6). The fear and embarrassment linked to stigmas often discourage patients from seeking help, which may explain why even their loved ones, sometimes, advise them to stop treatment to avoid being labeled. These stigmas can further lead to personal and family problems, and even cause the patients to neglect their illness. As a result, their condition may progress into a chronic disease (7). Stigmatizing behaviors, whether intentional or unintentional, are directed toward individuals perceived as different due to their gender, race, sexual orientation, illness, or other characteristics. Such behaviors can profoundly affect both public and individual health, resulting in emotional disorders, heightened stress, delays in accessing appropriate healthcare, and the premature discontinuation of medical treatments (8). Stigma arises from a combination of ignorance, prejudice, and discriminatory behavior toward a particular phenomenon. It involves a lack of understanding about the subject, negative attitudes, and biases that may, ultimately, lead to actions rooted in discrimination and misguided perceptions (9–12). Social stigma related to the Coronavirus Disease, known as COVID-19 stigma, imposes a burden on individuals socially, economically, and mentally (13). Previous research has underscored the significant influence of fear, hesitancy, and health literacy on public attitudes toward COVID-19 vaccination. A study conducted by Siewchaisakul et al. (2022) examined the relationship between vaccine literacy, fear, and hesitancy among village health volunteers in Thailand. The findings revealed that while vaccine literacy had a minimal impact on vaccine acceptance, fear and hesitancy notably diminished the willingness to receive the vaccine. The study highlighted that efforts to reduce misinformation and address psychological barriers were more effective than merely increasing knowledge. These results are consistent with broader research on COVID-19 stigma, indicating that fear and uncertainty often fuel stigmatizing attitudes (14). Public perceptions regarding COVID-19 prevention and vaccination significantly influence health outcomes, particularly among vulnerable populations. A study conducted by Wungrath et al. (2021) examined the knowledge, attitudes, practices, and vaccine acceptance among elderly individuals in Chiang Mai, Thailand. The results indicated that while awareness of COVID-19 transmission and prevention was high, vaccine hesitancy was still a concern, largely stemming from fears about potential side effects. Impressively, 100% of respondents expressed willingness to accept the vaccine if its efficacy were 80% or higher, underscoring the critical role of public trust and evidence-based health communication in shaping vaccine perceptions. These findings highlight the necessity for targeted public health strategies to combat fear, misinformation, and hesitancy, which are essential in addressing stigma-related perceptions of COVID-19 (15). Providing a vaccine, targeted treatment, or evidence-based care immediately following an outbreak poses significant challenges, as demonstrated by past experiences with emerging infections like Ebola hemorrhagic fever, severe acute respiratory syndrome, and Middle East Respiratory Syndrome. As a result, an outbreak of a pandemic infectious disease can evoke considerable fear and anxiety regarding the spread of infections (16–20). In early 2020, a social stigma emerged worldwide due to the COVID-19 pandemic (17). Social stigma can harm individuals together with their families, friends, and communities. The behavioral manifestation of stigma is discrimination, which can lead to community rejection and reduced opportunities for treatment and disease control (21). Healthcare professionals in 173 countries have encountered instances of bullying as a result of the stigma associated with COVID-19 (22). Four weeks into the COVID-19 pandemic's peak, 34.8% of the Chinese respondents experienced stigma or discrimination from people in other countries (23). In China, where COVID-19 first spread and later affected the world, it was found that there is a positive correlation between the stigma associated with COVID-19 directed at patients or their families and the symptoms of depression and financial stress (24). During the ongoing COVID-19 pandemic, various forms of stigma have directed towards afflicted individuals throughout the world, particularly of Asian descent, those with recent travel histories, and healthcare professionals. Notably, a singular incident of discrimination against a person with apparent Asian features occurred in Egypt, though it was met with widespread disapproval from the society, including the government officials (25). The stigma surrounding certain attributes affects not only the afflicted individuals (e.g., with COVID-19), but also those connected to them, including family members, service providers, and community members. This phenomenon, known as courtesy stigma, refers to the perceived and experienced stigma that associates from the general public (26). During the COVID-19 outbreak, Hubei Province was the hardest-hit region in China, with approximately 65,000 confirmed cases and around 3,000 deaths. The people of Hubei experienced both courtesy stigma and affiliate stigma due to their geographic association with the virus (27). The COVID-19 pandemic has heightened existing stereotypes against various groups. Before the national lockdown, a state of emergency was declared in Italy, yet daily life persisted. During this period, attitudes toward the Chinese community soured, their restaurants went largely empty, and many parents hesitated to send their children to schools with Chinese classmates (28). A study involving 260 healthcare workers (HCWs) in southern Italy examined the impact of stigma, job demands, and self-esteem on the frontline health providers for COVID-19 patients. The findings revealed that stigma greatly affected their outcomes and influenced compliance and management strategies related to pandemic risks (29). The high mortality rate of COVID-19 leads experts to consider it life-threatening and traumatic. They also believe that healthcare professionals and stigmatized groups are more likely to develop post-traumatic stress disorder (30). A study assessed the mental health of 894 HCWs in Iran during the COVID-19 pandemic and revealed a significant link between stigma perception and PTSD scores, including intrusion, avoidance, and hyper-vigilance. Stigma was notably higher among females, frontline HCWs, physicians, and medical residents, indicating their heightened psychological stress during this time (31). An investigation on the stigma faced by hospitalized COVID-19 patients in Lahore (Pakistan) using a modified HIV stigma scale and open-ended questions showed a widespread stigma, particularly regarding public attitudes and disclosure among the 140 interviewed patients. Key themes identified included social stigma and rejection, humiliating behavior from others, breaches of confidentiality, loss of trust and respect, and the impact of a COVID-19 diagnosis on their businesses (32). A study in Colombia investigated the association between stigmatization and fear of COVID-19 among 1,687 adults aged 18-76 years. The findings indicated that 34.1% of the participants reported high fear of COVID-19. It is worth noting that the general population exhibited significantly higher levels of stigma towards COVID-19 compared to HCWs. Additionally, there was a correlation between high fear of COVID-19 and stigma in 63.6% of the evaluated answers (33). A study in Russia assessed psychological distress and stigmatization among HCWs during the COVID-19 pandemic. It involved an online survey of 1,800 Russian-speaking healthcare professionals. The findings indicated that direct contact with COVID-19 increased stress levels, particularly among the younger and highly qualified specialists. Stigmatization was notably prevalent among the nurses and paramedical staff, though it was not directly linked to infection risk (34). The study in Tehran developed a questionnaire to assess COVID-19 stigma in the general population. Out of the 1,637 recruited citizens, 1,064 participated in interviews. The results indicated low levels of stigma, possibly due to social desirability, the widespread nature of the virus, or socio-cultural factors (35). A cross-sectional study in Delhi evaluated the prevalence of social stigma among frontline HCWs in the Department of Anesthesia and Critical Care during the COVID-19 pandemic. The findings revealed that over half of these workers experienced severe stigma, particularly related to public attitudes (36).

As mentioned, stigma negatively affects patients and their families and serves as a barrier to controlling the pandemic (37). Hence, understanding stigma and developing effective strategies to reduce it is highly important in every community, including the Iranian population (38). Stigma is a nuanced social phenomenon characterized by labeling, stereotyping, and discrimination, which ultimately leads to social isolation and a loss of status for those affected. In the context of COVID-19, stigma has been exacerbated by fear, misinformation, and public anxiety, resulting in negative attitudes toward individuals who are infected as well as healthcare workers. Within the realm of medical professionals, psychiatrists play a vital role in recognizing and addressing the psychological distress associated with stigma. Consequently, understanding their perspectives is essential for enhancing stigma reduction strategies in mental health care. While stigma has been extensively studied in various infectious diseases, such as Ebola and MERS, existing research has predominantly focused on its effects on the general population and frontline healthcare workers. Unfortunately, less attention has been given to psychiatrists, despite their essential role in addressing stigma-related psychological distress. As mental health specialists, psychiatrists not only engage with patients facing stigma but also play a significant role in shaping public health policies and strategies aimed at stigma reduction. Understanding their perspectives is crucial for enhancing stigma management within healthcare settings. This study seeks to address this oversight by examining psychiatrists’ attitudes toward COVID-19 stigma, a viewpoint that has been largely neglected in previous research. On the other hand, considering the pivotal role that psychiatrists play in preventing and addressing mental health complications linked to disease-related stigma, we have to first evaluate their attitudes toward the stigma potentially associated with COVID-19. As attitude encompasses beliefs, emotions, and behavioral intentions toward individuals or events (39), it is particularly essential to understand whether psychiatrists attitude toward the existence of such stigma and its related complications. This study attempts to investigate psychiatrists' perspectives on the stigma surrounding COVID-19, which could guide the development of effective interventions. The findings will aid mental health professionals in determining whether to create preventive and therapeutic strategies aimed at mitigating the psychological impact resulting from this stigma.

## 2 Materials and methods

### 2.1 Setting and procedure

This study was a cross-sectional survey using a researcher-made questionnaire to collect data from psychiatrists practicing in Tehran. The present research utilized a multistep approach to develop, validate, and assess the reliability of COVID-19 Stigma Attitude Scale for Psychiatrists (CSASP) designed to measure psychiatrists' attitude toward the stigma associated with COVID-19. The research was conducted in Tehran City (Iran) between April 9, 2023 and May 26, 2023.

### 2.2 Questionnaire content, validity and reliability

We established an initial pool of related and relevant items through an extensive literature search utilizing PubMed and Google Scholar. At that time, our review revealed no existing Persian or English questionnaires specifically addressing COVID-19-related stigma. Consequently, we incorporated evidence regarding the stigma associated with SARS and HIV/AIDS into our search, extracting items from the SARS- and HIV/AIDS-related questionnaires to expand our pool. To conduct this study, we developed a custom questionnaire to assess the stigma associated with COVID-19. The original questionnaire consisted of 21 questions, each utilizing a five-point Likert scale that ranged from 5 to 1 (strongly agree, agree, no opinion, disagree, strongly disagree). In the initial phase, the questionnaire was shared with 10 psychiatrists, who are specialists in the area. They were requested to provide their feedback on the relevance, clarity and comprehension, simplicity, and the essential nature of each item. They were further asked to evaluate each item using a four-point ordinal rating scale to assess relevance (1 = not relevant, 2 = somewhat relevant, 3 = quite relevant with minor revisions, and 4 = highly relevant); clarity and comprehension (1 = not clear and understandable, 2 = somewhat clear and understandable, 3 = quite clear and understandable with minor revisions, and 4 = very clear and understandable); and simplicity (1 = not simple, 2 = somewhat simple, 3 = quite simple with minor revisions, and 4 = very simple). Additionally, they rated each item on a three-point scale to gauge essentiality (1 = not beneficial and not essential, 2 = beneficial but not essential, and 3 = beneficial and essential).

In continuation, the content validity index (CVI) was determined using the relevancy measure. Specifically, the item content validity index (I-CVI) for each item was calculated by dividing the number of experts, who rated the item as highly relevant by the total number of experts. An item was deemed relevant if the I-CVI was 0.78 or higher. The I-CVI values range from 0 to 1; values closer to 1 indicate greater relevance. The overall content validity of the questionnaire tool, referred to as the scale content validity index (S-CVI), was evaluated as the average of I-CVIs. The questionnaire was considered valid if the S-CVI met or exceeded 0.90 (40–42). We utilized the essentiality measure to calculate the item content validity ratio (I-CVR) using Lawshe’s formula as follows:


CVR=[(E-(N / 2)/(N / 2))]

Where, E represents the number of panelists, who rated the item as “beneficial and essential, and N denotes the total number of panelists. The I-CVR values range from 0.1 to 1, with values closer to 1 indicating that the item is deemed more essential. An I-CVR of at least 0.78 is necessary to classify an item as essential. If the value falls between 0.70 and 0.78, the item requires revision, and for values below 0.70, the item should be eliminated (41). To determine the Item Face Validity Index (I-FVI) for each item, ten raters were asked to independently evaluate each item based on the criteria of clarity, comprehension, and simplicity. The I-FVI for each item was calculated by dividing the number of raters, who assigned a clarity, comprehension, and simplicity rating of 3 or 4 by the total number of raters for that item. An item is considered clear, understandable, and simple if the I-FVI value is equal to or greater than 0.78. The Scale Face Validity Index (S-FVI) is established by averaging the I-FVI scores across all items in the scale. To validate the FV of the questionnaires, at least 83% of the experts must express their approval (43). In this study, the statistical population consisted of all psychiatrists in Tehran City in 2023. Among the 817 psychiatrists in the city, the sample size was determined according to the differences between the two sexes using G POWER software. With an effect size of 0.5, which is classified as medium to large, and a study power (1 −β ) of 0.8 at a significance level of α= 0.05, the calculated sample size amounted to 64 individuals in each group. This means that the sample included 64 women and 64 men. For the purposes of this study, attitudes were measured quantitatively. Overall, 131 psychiatrists from Tehran were randomly selected from April 9 to May 26, 2023, with a roughly equal distribution of genders (retrieval rate:87.3%). A non-probability convenience sampling technique was used to recruit psychiatrists in Tehran. The survey was conducted both online and in print format, distributed in psychiatric hospitals and clinics. The participants voluntarily responded to the researcher-made questionnaire designed to gather demographic information, including age, gender, marital status, work history, workplace setting, and experiences related to treating or consulting patients with COVID-19. The psychiatrists involved in validating the questionnaire were excluded from the final study sample to avoid response bias. The collected data were analyzed confidentially. To ensure the confidentiality of participants, all data collection procedures followed ethical guidelines. Before participating, respondents were informed that their answers would remain anonymous and confidential, and that no personal identifiers (such as names, contact details, or IP addresses) would be collected. To evaluate the internal consistency of the questionnaire, we computed Cronbach’s alpha based on the responses of all the 131 participants. Additionally, we calculated the corrected item-total score correlation coefficient, and the Cronbach’s alpha value was determined for each item if it was removed from the analysis. An item-total correlation value exceeding 0.2 was deemed a good indicator of discrimination (44). A Cronbach’s alpha value greater than 0.6 was deemed acceptable for confirming internal consistency (45, 46). The experts assessed both the content and face validity of the questionnaire. Almost all items achieved I-CVI, I-CVR, and I-FVI values >0.78, with the exception of six questions that fell short of the necessary standards for content validity and were, subsequently, removed from the questionnaire. As a result, the total number of questions in the questionnaire was reduced to 15. So the distributed questionnaire consisted of 15 questions, scoring from 15 to 75 points, with higher scores indicating a greater level of attitudes towards the existence of COVID-19 stigma. As a measure of the questionnaire's effectiveness, the S-CVI and S-FVI values were recorded at 0.90 and 92.6%, respectively. To assess the reliability of the 15-question questionnaire, Cronbach's alpha was computed using the SPSS software (ver. 25). An alpha value above 0.7 is considered acceptable, while a value above 0.8 is deemed appropriate. The Cronbach's alpha obtained for the 15-question questionnaire was 0.861, indicating it falls within the appropriate range. Additionally, the corrected item-total correlation was calculated for all questions, and all the values were found to be equal to or above 0.2 (in the acceptable range), and thus, confirming that all questions can be utilized.

### 2.3 Attitude of psychiatrists towards the presence of stigma about COVID-19

To analyze psychiatrists' attitudes toward COVID-19 stigma, we first calculated the average attitude score. Based on the distribution of scores, attitudes were categorized using standard deviation (SD) thresholds as follows: Weak Attitude (≤ -1 SD), Rather Weak Attitude (Between -1 SD and Mean), Rather Strong Attitude (Between Mean and +1 SD), Strong Attitude (≥ +1 SD). To evaluate the normality of our data, we analyzed the Skewness and Kurtosis values. The findings indicated a Skewness of -0.708, which falls within the acceptable range of -1 to +1, signifying minimal asymmetry in the data distribution. The Kurtosis value was 0.305, also within the acceptable range of -1 to +1, suggesting that there are no significant deviations concerning the distribution's peakness or flatness. Given that our sample size exceeds 30, the Central Limit Theorem suggests that the sampling distribution approaches normality. Therefore, we proceeded with parametric tests (t-test and ANOVA) for group comparisons. Descriptive statistics (mean, standard deviation, and frequency distribution) were used to summarize the responses. Independent sample t-tests and one-way ANOVA were applied to examine differences in stigma perceptions based on demographic variables.

## 3 Results

### 3.1 Demographic characteristics of the participants

The demographic characteristics of the psychiatrists in the study are summarized in **Table 1**. Overall, 67 participants (51.1%) were female and 64 participants (48.9%) were male. The average age of the subjects was 46.65 ± 9.6 years. A total of 37 participants (28.2%) were identified as unmarried, and the average work experience the surveyed participants was 14.51 ± 9.05 years. In terms of workplace setting, 58 individuals (44.3%) were employed in hospitals or clinics, 35 individuals (26.7%) worked in private offices, and 38 individuals (29%) practiced in both environments. Furthermore, 70 individuals (53.4%) were engaged in the treatment or counseling of patients with COVID-19.

**Table 1:** Sociodemographic and occupational characteristics of the psychiatrists.

Variable	Participants (N=131)
Gender N (%)	
Male	64 (48.9%)
Female	67 (51.1%)
Age (years) (M ± SD)	46.65 ± 9.6
Marital status N (%)	
Married	94 (71.8%)
Unmarried	37 (28.2%)
Work experience (years) (M ± SD)	14.51 ± 9.05
Workplace setting N (%)	
Hospitals or clinics	58 (44.3%)
Private offices	35 (26.7%)
Both environments	38 (29%)
Treatment or counseling of patients with COVID-19 N (%)	
Engaged	70 (53.4%)
Not engaged	61 (46.6%)

M, Mean; SD, Standard Deviation.

### 3.2 Attitude of psychiatrists towards the presence of stigma about COVID-19

The average score of psychiatrists’ attitude towards COVID-19 stigma in Tehran was found to be 51.16 ± 8.83. The highest score was 68 and the lowest was recorded as 25. The results revealed that 19 individuals (14.5%) exhibited a weak attitude, 41 individuals (31.3%) displayed a rather weak attitude, 54 individuals (41.2%) showed a rather strong attitude, and 17 individuals (13%) demonstrated a strong attitude toward the presence of COVID-19 stigma (**Table 2**).

**Table 2:** Attitude of psychiatrists towards the presence of stigma about COVID-19.

Variable	Participants N (%)
Prevalence	
Weak attitude	19 (14.5%)
Rather weak attitude	41 (31.3%)
Rather strong attitude	54 (41.2%)
Strong attitude	17 (13%)
Average score	51.16 ± 8.83

In this study, there was no significant difference in attitude toward COVID-19-associated stigma according to age, gender, marital status, work experience, workplace, treatment, or non-treatment of people with COVID-19 (**Table 3**).

**Table 3:** Relationship between demographic variables and psychiatrists' attitude towards the stigma associated with COVID-19.

Variable	Average score (Mean ± SD)	*P* -Value
Gender*		
Male	50.20 ± 9.99	0.223
Female	52.08 ± 7.52	
Marital status*		
Married	51.51 ± 8.54	0.481
Unmarried	50.29 ± 9.61	
Workplace setting**		
Hospitals or clinics	50.96 ± 8.57	0.520
Private offices	52.54 ± 9.74	
Both environments	50.21 ± 8.42	
Treatment or counselling of patients with COVID-19*		
Engaged	50.97 ± 8.70	0.786
Not engaged	51.39 ± 9.05	
Age (years)**		
Age ≤39	50.32 ± 8.75	0.567
39< Age ≤ 45	50.85 ± 7.24	
45< Age ≤ 54	52.86 ± 8.95	
Age >54	50.31 ± 9.95	
Work experience (years)**		
W.E. ≤ 7	50.29 ± 9.13	0.796
7< W.E. ≤ 13	52.00 ± 7.16	
13< W.E. ≤ 20	52.03 ± 9.42	
W.E. > 20	50.57 ± 9.62	

SD, Standard Deviation; W.E., Work Experience; *Independents sample T-test; **One-way ANOVA.

Upon examining the survey responses, it was found that 96 individuals (73.3%) agreed with the existence of stigma surrounding COVID-19 at the onset of the pandemic. Out of these 96 individuals, 11 individuals (11.5%) disagreed with the presence of this stigma currently, 18 individuals (18.7%) declared that they have no opinion on the matter, and the rest (67 individuals, 69.8%) still maintained their belief in the existence of stigma surrounding COVID-19.

In the present study, the psychiatrists' attitudes towards two other medical disorders (AIDS and leprosy) were also investigated. The results showed that a total of 83 respondents (63.3%) agreed on the presence of stigma related to AIDS and leprosy. Moreover, 53 (63.8%) of these 83 respondents confirmed their belief in the presence of stigma associated with COVID-19.

## 4 Discussion

We designed a 15-question Persian questionnaire regarding the psychiatrists’ attitude towards COVID-19-assiciated stigma in Tehran City and its validity and reliability were confirmed. The findings of this study revealed that the average attitude score of psychiatrists toward COVID-19 stigma in Tehran was 51.16 ± 8.83, with scores ranging from 25 to 68. Based on standard deviation classifications, 19 psychiatrists (14.5%) exhibited a weak attitude, 41 (31.3%) displayed a rather weak attitude, 54 (41.2%) demonstrated a rather strong attitude, and 17 (13%) showed a strong attitude toward the presence of COVID-19 stigma. Additionally, among the 131 psychiatrists surveyed, 69.8% acknowledged the existence of COVID-19 stigma, while 11.5% believed that stigma had diminished, and 18.7% remained neutral. When comparing attitudes toward stigma in other infectious diseases, 83 participants recognized stigma associated with AIDS and leprosy, with 53 of them also perceiving stigma toward COVID-19. These findings offer valuable insights into the varying perceptions of stigma among psychiatrists, which can be further contextualized by comparing them with the findings from other relevant studies. They further reveal the persistent perceptions of stigma, which carry several critical implications. Moreover, the research results indicate that psychiatrists, who are mental health specialists, recognize the existence of not only COVID-19-associted stigma but also for other historically stigmatized conditions like AIDS and leprosy. This highlights their awareness of stigma as both a psychological and societal issue. The acknowledgment of stigma by psychiatrists shows that they are in a unique position to address its consequences such as patients avoiding care, developing anxiety, or experiencing social isolation. This places psychiatrists at the forefront of anti-stigma efforts. It is worth noting that 69.8% of the participants maintained their belief in the ongoing stigma associated with COVID-19, suggesting that stigma remains an unresolved issue even as public health measures evolve. Such stigma can worsen mental health outcomes, delay treatment-seeking behavior, and undermine trust in healthcare systems. Individuals who face stigma may refrain from seeking medical care due to concerns about social exclusion, resulting in delays in diagnosis and treatment. This phenomenon has been observed during previous pandemics, where stigma led to underreporting of symptoms and non-compliance with health guidelines, thereby increasing transmission rates of the disease. Furthermore, stigma undermines trust in healthcare institutions. Misinformation, combined with fear-driven narratives, can foster distrust in medical advice and vaccine hesitancy, ultimately diminishing public adherence to preventive measures (47, 48). This erosion of trust can have long-term ramifications, as individuals may become hesitant to engage with healthcare services even after the pandemic, exacerbating existing health disparities. Persistence of stigma allows healthcare policymakers to implement targeted anti-stigma campaigns and educational programs aimed at reducing its impact. The overlap in stigma perceptions for AIDS, leprosy and COVID-19 (with 53 out of 83 respondents indicating shared views) underscores common societal drivers like fear of contagion, misinformation, and historical biases. By understanding that stigma dynamics are similar across the mentioned infectious diseases, this study provides a foundation for cross-disease stigma-reduction strategies, which may be more sustainable and effective than those focused on a single disease. Our findings showed no significant differences in the psychiatrists’ stigma attitudes based on age, gender, marital status, work experience, or workplace, suggesting that stigma is a universal issue among psychiatrists, cutting across demographic boundaries. Interventions aimed at reducing stigma should, therefore, focus on system-wide education rather than targeting specific subgroups. Accordingly, since psychiatrists play a crucial role in mental health care, especially during pandemics when stigma can exacerbate anxiety, depression and trauma, the present research emphasizes the importance of involving them in anti-stigma education and advocacy. Furthermore, by equipping psychiatrists to identify and address stigma, healthcare systems can better support patients and communities.

Faghankhani et al. examined the levels of stigma within the non-infected general population in Tehran City. Utilizing a validated stigma scale, they found that 86.8% of the participants exhibited low levels of stigma, while 13.2% displayed moderate stigma, with no participants reporting severe stigma. In contrast, our findings revealed that a higher proportion of the participants (psychiatrists) exhibited strong or rather strong stigma-related attitudes, totaling 54.2% combined. Only 14.5% of them demonstrated weaker attitudes toward stigma. This discrepancy underscores significant differences between healthcare professionals and the general population. Although psychiatrists, due to their specialized knowledge, may have a better understanding of the psychological effects of stigma, they remain susceptible to its influence. Conversely, as suggested by Faghankhani et al., the general population may report lower levels of stigma as a result of widespread exposure to COVID-19 in large cities like Tehran, which may have contributed to the normalization of the pandemic experience. Additionally, social desirability bias could affect response patterns, leading individuals to provide socially acceptable answers to mitigate the risk of judgment (35).

In Jain et al.’s study at a tertiary care hospital in Delhi, 56.6% of the frontline HCWs reported experiencing severe stigma, which is significantly higher than the 13% of psychiatrists who expressed strong stigma-related attitudes in the present research. Jain et al. found that the highest levels of stigma were associated with concerns regarding public perception. Additionally, factors such as age (particularly those over 30), male gender, and lower educational attainment were linked to increased stigma levels. In contrast, our study revealed a wider distribution of attitudes among psychiatrists, with 41.2% showing rather strong stigma-related attitudes and 14.5% exhibiting weak attitudes. This may be attributed to the psychiatrists' professional training in mental health, which equips them with better-coping strategies and a deeper understanding of the psychological effects of stigma. Jain et al. specifically focused on frontline HCWs, including anesthetists, ICU staff, and nurses, who faced direct exposure to COVID-19 patients, leading to greater stigma due to fears of infection and public discrimination. In comparison, psychiatrists may have faced less societal scrutiny due to their more indirect involvement. Moreover, public awareness campaigns and increased familiarity with COVID-19 in Tehran may have contributed to a reduction in intensely stigmatizing attitudes, unlike the elevated stigma levels reported by frontline HCWs in Delhi (36).

Sawaguchi et al. examined COVID-19-associated stigma within the general population in Japan using an adapted version of the Cancer Stigma Scale. They identified five key factors contributing to COVID-19 stigma: Avoidance, Personal responsibility, Severity, Policy opposition, and Awkwardness. Notably, their findings revealed that COVID-19 stigma scores were higher among the participants aged 70 and older, particularly in the Avoidance and Awkwardness subscales. In contrast, the present research psychiatrists, a younger and more educated group, demonstrated that stigma was influenced more by their professional experiences rather than age-related fears. Unlike Sawaguchi et al.'s population-based sample, the awareness of stigma among psychiatrists may be shaped by their professional background and expertise in mental health, resulting in a more varied range of attitudes. Additionally, their study indicated that heightened societal fears about infection and its repercussions contributed to Severity being identified as the most significant stigma factor. Although Tehran’s psychiatrists encounter lower societal stigma, their perception of stigma may still be increased by patient experiences and broader dynamics within the healthcare system (13).

When comparing the findings of Duan et al. conducted in Hubei Province (China) with ours, notable differences in COVID-19 stigma perception become apparent. Duan et al. categorized the participants into three stigma profiles using Latent Profile Analysis: a) Denier (35.98%), which reflects low levels of perceived courtesy and affiliate stigma; b) Confused Moderate (48.13%), indicating moderate levels of stigma; and c) Perceiver (15.89%), representing high levels of perceived stigma. While 15.89% of the participants in Hubei experienced high stigma levels ("Perceiver"), only 13% of the psychiatrists in Tehran reported strong stigma-related attitudes; however, 41.2% of Tehran psychiatrists expressed rather strong attitudes, suggesting a broader moderate sensitivity to stigma comparable to the Confused Moderate group (48.13%) in the Hubei study. Given that psychiatrists are highly educated professionals trained in mental health, this may contribute to a heightened sensitivity toward stigma. In contrast, Duan et al. identified education and perceived threats as risk factors for high stigma perception within the general population of Hubei. The study highlighted how geographic associations with COVID-19 contributed to courtesy stigma, with terms like "Wuhan virus" exacerbating rejection and exclusion. Psychiatrists in Tehran, however, may encounter less region-specific stigma, with a greater emphasis on the psychological impact of the pandemic and societal attitudes. Duan et al. found that the “Perceiver” group participants were more likely to view COVID-19 as severe and threatening, a perception that aligns with stigma drivers on a global scale. In our study, although direct perceptions of severity were not measured, the pronounced attitudes among psychiatrists suggest an increased awareness of the consequences of stigma within the healthcare system. Media exposure and engagement were significant predictors of stigma in the Hubei study. Conversely, the psychiatrists in our Tehran study might have experienced stigma more through professional exposure rather than media influences, leading to more nuanced yet pronounced attitudes (27).

In comparison to Ramaci et al.’ study in southern Italy with ours, several distinguished similarities and differences emerge. In addition to measuring psychological demands and self-esteem, Ramaci et al. assessed the effect of stigma on 273 HCWs in terms of discrimination, fear, and burnout. As a key finding, they found that “stigma discrimination” had a mean of 1.57 ± 0.72, and “stigma fear had a mean of 2.88 ± 0.89. Furthermore, higher levels of “burnout” were reported among the female HCWs and those with long-term contracts. The study identified stigma as a significant predictor of burnout ( β= 0.317, p< 0.001) and fatigue ( β= 0.248, p< 0.001). While Ramaci et al. concentrated on burnout and psychological strain, our study emphasized the prevalence of stigma perceptions within a specialized group of healthcare professionals (i.e. psychiatrists). Although Ramaci et al. highlighted stigma as a contributor to burnout and fatigue among a group of frontline HCWs, our findings indicated that stigma is also present among psychiatrists, albeit at lower levels compared to global frontline HCWs. Psychiatrists may experience stigma indirectly, mainly through secondary exposure to the psychological consequences observed in their patients (29).

The findings of Chew et al.’s longitudinal study of residents in training across various specialties in Singapore offer valuable insights into stigma levels among healthcare professionals in two unique contexts. They assessed stigma among HCWs using the Healthcare Workers Stigma Scale (HWSS). The research involved 274 residents at the baseline and 221 residents at a three-month follow-up. At baseline, the mean total score of HWSS was 22.5 with a standard deviation of 6.84, while the follow-up score showed a slight decrease to a mean of 20.8 with a standard deviation of 6.92. Particularly noteworthy was the decrease in scores related to the Disclosure Concerns subscale from 6.27 ± 2.29 to 5.80 ± 2.37, as well as a decline in public attitudes from 6.61 ± 2.28 to 5.95 ± 2.26. These findings suggest a positive trend in reducing stigma among the residents over the three-month period. Chew et al. noted an initial presence of stigma among the residents, which was decreased over time, likely due to nationwide awareness campaigns and public support for HCWs in Singapore. In agreement with our findings, the specialized knowledge of psychiatrists regarding mental health may heighten their sensitivity to the psychological impact of stigma. Additionally, Chew et al. found that perceived stigma was correlated with higher levels of traumatic stress and avoidance coping mechanisms, factors that could similarly influence the stigma attitudes of psychiatrists in Tehran (49).

When comparing our results with a similar research conducted by Zandifar et al. in Iran, which focused on HCWs, we can identify both significant similarities and differences. Zandifar et al. examined the prevalence of stigma and post-traumatic stress symptoms (PTSSs) among HCWs exposed to COVID-19 patients across nine hospitals in Alborz Province, Iran. The findings indicated that stigma was notably more prevalent among female HCWs ( p = 0.01), frontline HCWs ( p = 0.006), and physicians ( p < 0.04), orderly.

The correlation analysis revealed a strong association between stigma and PTSS scores (coefficient: 0.83). In contrast, 13% of the psychiatrists in our study reported strong stigma attitudes, with 41.2% displaying rather strong attitudes. While Zandifar et al.’s study indicated elevated stigma among frontline HCWs, the specific categorization of this stigma as strong or moderate was not explicitly provided, making direct numerical comparisons difficult. The higher levels of PTSS reported by frontline HCWs in Zandifar et al.’ study are likely attributable to their direct exposure to COVID-19 patients, which intensifies both psychological stress and perceptions of stigma. Conversely, the psychiatrists in our study, though not frontline HCWs, may indirectly encounter stigma through their professional experiences and by observing the mental health impacts on their patients. Zandifar et al. highlighted a significant relationship between stigma and PTSS, underscoring the psychological toll that stigma inflicts. Although our study did not measure PTSS, the relatively high percentage of “rather strong” stigma attitudes (41.2%) suggests a considerable sensitivity to the effects of stigma within healthcare systems and the broader society (31).

Our findings were compared with those of Imran et al.’s study, which investigated the stigma experienced by hospitalized COVID-19 patients in Lahore (Pakistan). This comparison offers valuable insights into how stigma manifests among healthcare professionals in Tehran and quarantined COVID-19 patients in Lahore. Imran et al. (2020) examined stigma among 114 COVID-19 patients in a tertiary care hospital using a modified HIV stigma scale. The results gave the following mean scores: concerns about public attitudes (7.43 ± 1.43), disclosure concerns (6.89 ± 1.45), personalized stigma (6.82 ± 1.28), and negative self-image (6.72 ± 1.34). Moreover, their study highlighted widespread stigma, with patients reporting social rejection, breaches of confidentiality, and humiliating behavior. In contrast, our study found that 54.2% of the psychiatrists reported strong or rather strong stigma attitudes, reflecting their professional awareness and societal perceptions of stigma. In Imran et al.'s study, social rejection was a primary theme, where the patients faced humiliation, ostracization, and loss of respect due to their COVID-19 diagnosis. The psychiatrists in our study reported stigma at an attitudinal level under the influence of their professional roles and understanding of the mental health consequences of stigma.

The Pakistani COVID-19 patients expressed heightened concerns about disclosure, with a mean score of 6.89 ± 1.45, and many of them feared being labeled as “contagious” or “dirty.” Although we did not directly measure disclosure concerns among the psychiatrists, it is likely that they similarly perceive stigma due to their roles in counseling stigmatized patients. The stigma experienced by patients in Imran et al.'s study reflects cultural and societal reactions to infectious diseases in Pakistan, where families faced labeling and social rejection. In contrast, psychiatrists may view stigma through a professional lens, which could increase their sensitivity to the issue while reducing their personal experiences of rejection. Both studies highlight the impact of public attitudes on stigma. Furthermore, the Pakistani participants reported experiences of verbal abuse, breaches of confidentiality, and economic impacts such as business losses resulting from community labeling. These findings underscore the societal barriers to addressing stigma in low- and middle-income countries (LMICs) (32).

Cassiani-Miranda et al.'s study examined the stigma associated with COVID-19 among 1,687 adults in Colombia, focusing on both the general population and HCWs. Their findings provide crucial insights into the nature of stigma across different demographics. The study revealed that 42.3% of the general population and 35.4% of HCWs believed that foreigners posed a higher risk of transmitting COVID-19. Additionally, 56.1% of the general population and 48.1% of HCWs attributed the virus's spread to irresponsible behavior. Notably, 12.4% of the general population and 5.8% of HCWs felt that they should be isolated because of their contact with COVID-19 patients. While the Colombian study highlighted stigma particularly linked to fears surrounding foreigners and healthcare professionals, the findings from Tehran reflect a more nuanced awareness of professional stigma rather than direct stigmatization. In Colombia, the fear of foreigners emerged as a significant factor driving stigma (42.3%), whereas Tehran psychiatrists exhibited stigma attitudes shaped more by their professional knowledge and societal perceptions. This contrast underscores how cultural and social contexts can influence the expression of stigma. Furthermore, in Colombia, 12.4% of the general population believed that HCWs should be isolated due to their exposure to COVID-19 patients, which aligns with findings in other regions where HCWs were seen as potential disease carriers. Although Tehran psychiatrists are not frontline HCWs, they likely possess a heightened awareness of stigma-related impacts through their professional lens. Moreover, Cassiani-Miranda et al. found that 56.1% of the general population attributed COVID-19 infections to irresponsible behavior, reflecting a stigma rooted in moral judgments. Our study, while not explicitly addressing these attributions, observed significant stigma attitudes among psychiatrists, which may be influenced by similar societal biases (33).

Our findings were analyzed in conjunction with the findings of Baldassarre et al., who conducted a comprehensive examination of stigma and discrimination (SAD) due to the COVID-19 pandemic on a global scale. They emphasized that stigma surrounding SARS-CoV-2 is influenced by several interconnected factors, including a lack of knowledge, fear of infection, and societal biases. While they did not provide explicit quantification of stigma prevalence, they noted that frontline HCWs globally faced more severe stigma due to their visible roles in the pandemic response. In contrast, in our study, 54.2% of the psychiatrists reported strong and rather strong attitudes toward stigma. This highlights an increased sensitivity to the impact of stigma by psychiatrists. In other words, though psychiatrists are not directly involved in frontline treatment, they often address the psychological effects of stigma among their patients. This distinction underscores the different nature of stigma exposure, which contributes to the variance in findings. Baldassarre et al. pointed out that stigma often emerges from knowledge gaps and fear-driven behaviors, particularly during pandemics. However, the psychiatrists in our study exhibited a heightened awareness of the psychological toll of stigma, which may account for the significant proportion of them (41.2%) expressing rather strong attitudes (19).

This study evaluated psychiatrists' attitudes toward COVID-19 stigma in Tehran and found no significant differences based on age, gender, marital status, work experience, workplace, or involvement in treating COVID-19 patients. Research findings from various other regions worldwide (e.g. Pakistan) suggest that being male is independently associated with higher stigma scores. For instance, Jain et al. found that male HCWs had higher stigma levels across all subscales, including personalized stigma and public attitudes (36). In contrast, our study revealed no significant differences between male and female psychiatrists, signifying that professional training may equalize their perceptions of stigma. Several studies have shown that younger age groups (under 30) are more likely to report stigma-related concerns like negative self-image and worries about disclosure. For example, frontline HCWs under 30 reported a higher fear of stigma compared to older participants (36). However, our study found that age had no impact on stigma attitudes, potentially due to the psychiatrists' shared awareness of mental health implications across different age groups. Marital status and work experience have been identified as contributing factors in some studies (e.g. Zandifar et al.), where married HCWs exhibited greater PTSSs linked to stigma (31). Similarly, frontline HCWs with fewer years of experience reported higher stigma levels in some regions (36). Nevertheless, our study did not find any significant correlation in this regard, highlighting the uniformity of psychiatrists’ attitudes, regardless of marital status or professional experience. Multiple studies emphasize that frontline HCWs in hospitals or ICUs experienced significantly higher stigma compared to those working in non-treatment environments (29, 36). For instance, Baldassarre et al. reported that HCWs in direct patient contact perceived greater stigma due to the fear of contagion (19). In contrast, the psychiatrists in our study, (whether involved in treatment or not) showed no significant differences in their attitudes toward stigma. This finding may reflect the indirect role of psychiatrists in pandemic care that considerably protects them from personal experiences of stigma.

This study found that 96 individuals initially acknowledged the existence of stigma surrounding COVID-19 at the onset of the pandemic. Of these, 67 individuals (69.8%) continued to believe in the presence of stigma, while 11 individuals (11.5%) disagreed with its persistence, and 18 individuals (18.7%) expressed neutrality. These findings highlight a changing perception of stigma over time. Comparisons with other studies revealed important trends and differences in the persistence, reduction, and ambivalence of stigma. For instance, Imran et al. reported that stigma continued to affect COVID-19 patients in quarantine due to social rejection and labeling, despite the passage of time (32). Similarly, Zandifar et al. found that HCWs continued to experience stigma long after the initial surge of cases, primarily driven by fears of infection and professional exposure (31). The 69.8% of participants in our study, who still believe in the existence of stigma, closely align with these findings, indicating that stigma is resilient and not easily eliminated without targeted interventions. Additionally, Baldassarre et al. reported gradual reductions in stigma as knowledge and public awareness about COVID-19 increased. However, stigma reduction was more significant in populations that had robust public health campaigns (19). In our study, 11.5% of the participants disagreed with the current presence of stigma, reflecting a small but measurable decline. This aligns with a global trend of stigma reduction among individuals who gained confidence in the management and prevention strategies of the pandemic. The finding that 18.7% of the participants expressed no opinion results from the studies that identified neutral or uncertain attitudes toward COVID-19 stigma. For example, Cassiani-Miranda et al. found that a subset of the general population remained unsure about stigma's impact, likely due to indirect exposure to stigma or limited understanding of its psychological consequences (33). Chew et al. also reported that some healthcare trainees displayed ambivalence toward stigma as they struggled to reconcile their personal fears with professional responsibilities (49). In the present study, the neutral responses may reflect conflicting experiences or a lack of direct exposure to stigma, resulting in ambivalence. The persistence of stigma among 69.8% of the participants aligns with the global findings that stigma often becomes entrenched due to fear of contagion and misinformation. Historical parallels can be drawn with prior pandemics such as HIV and SARS, where societal rejection of individuals associated with the disease was prevalent (e.g. Imran et al.) (32). The 11.5% reduction in stigma perception observed in our study may be attributed to increased public education and awareness campaigns, widespread vaccination programs that reduced fear of the virus, and shifts in societal narratives that normalized the pandemic’s impact, as seen in Baldassarre et al.’s findings (19). As mentioned, in the present research, 18.7% of the participants provided neutral responses; One possible explanation lies in the principle of clinical impartiality, where psychiatrists may emphasize an objective, evidence-based approach instead of articulating strong opinions on socially sensitive matters. This neutrality may arise from a commitment to maintaining professional boundaries and avoiding biases that could impact patient care. It has been suggested that upholding neutrality in psychiatric practice can help reduce bias and foster patient-centered care. Additionally, some psychiatrists may view stigma as a societal issue rather than a clinical one, causing hesitation in addressing its presence and impact. Another possible explanation lies in the uncertainty surrounding the long-term trajectory of COVID-19 stigma. As stigma evolves in response to public perception, media influence, and government policies, some psychiatrists may perceive it as neither entirely persistent nor wholly resolved. This leads them to adopt a more neutral stance. The present study found that 83 respondents acknowledged the existence of stigma related to AIDS and leprosy, with 53 of them also expressing their belief in the stigma associated with COVID-19. This result provides an opportunity to examine stigma as a recurring theme across infectious or chronic diseases as highlighted in the literature. Stigma toward HIV/AIDS has historically been one of the most enduring and well-documented forms of disease-related stigma, stemming from societal fears, moral judgments, and misinformation. Herek et al. emphasized that individuals living with HIV/AIDS often experience social isolation and discrimination due to unfounded moral biases (50). Logie et al. noted similarities between the stigma surrounding AIDS and COVID-19, particularly due to the role of fear and misinformation in fostering the rejection of affected individuals (51). In our study, 53 respondents recognized stigma across all the three diseases (i.e. COVID-19, AIDS, and leprosy), implying that stigma may be driven by similar societal dynamics, regardless of the specific characteristics of the diseases. The overlap observed in our findings, where 53 individuals acknowledged stigma across these three diseases, suggests shared underlying factors, which include fear of contagion that is common to COVID-19, HIV/AIDS, and leprosy. Misinformation and lack of education that have been documented as significant contributors to stigma across pandemics. Historical and cultural biases regarding the stigmas associated with AIDS and leprosy, may also influence the perceptions of COVID-19-associated stigma.

The stigma surrounding infectious diseases, including COVID-19, is significantly shaped by cultural norms and societal values, which in turn influence perceptions and behaviors towards those affected. In collectivist societies, such as those found in East Asia and the Middle East, there is a strong focus on community cohesion and social harmony. Consequently, diseases are often viewed as threats to the collective well-being, resulting in social ostracism and discrimination against infected individuals. For example, during the COVID-19 pandemic, many people in certain communities experienced verbal and physical harassment due to fears and misconceptions regarding the transmission of the virus (52). In contrast, individualistic cultures, such as those common in Western Europe and North America, tend to emphasize personal responsibility and autonomy. In these contexts, stigma can manifest through blame and moral judgments directed at individuals who contract the disease, often linking illness to personal shortcomings or irresponsible behavior. This viewpoint can result in discrimination and social distancing, serving not only as a health precaution but also as a means of social exclusion. Historical contexts significantly influence the formation of stigma. For instance, in India, conditions such as leprosy have historically been linked to ideas of divine punishment and impurity, resulting in the enduring stigmatization of those affected. Research indicates that individuals with leprosy and HIV/AIDS in Southern India face considerable restrictions on social participation due to stigma, which adversely impacts their quality of life and mental health (53). Comprehending cultural nuances is vital for creating effective stigma-reduction interventions. In collectivist societies, strategies that involve community leaders and foster collective empathy tend to be more impactful, whereas in individualistic cultures, the focus on personal education and confronting moralistic judgments may be more critical. Acknowledging the cultural foundations of stigma enables the development of tailored public health approaches that align with the values and beliefs of specific populations, ultimately enhancing their effectiveness.

The attitudes of psychiatrists towards COVID-19 stigma can be understood through established psychological theories. Goffman’s Stigma Theory (1963) characterizes stigma as a “spoiled identity” that results in social devaluation, which can affect psychiatrists’ perceptions of individuals who are stigmatized (54). Labeling Theory suggests that societal labels shape self-identity and behavior, potentially explaining why some psychiatrists may be hesitant to take a strong position against COVID-19 stigma. Social Identity Theory indicates that psychiatrists, as members of the medical community, may experience cognitive dissonance when stigma conflicts with their professional roles, leading to neutral or ambivalent attitudes. Additionally, Attribution Theory posits that psychiatrists’ perceptions are influenced by whether they attribute COVID-19 infections to personal irresponsibility or external factors, which in turn affects the intensity of the stigma. Understanding these theoretical frameworks is crucial for developing effective strategies to reduce stigma within psychiatric practice and healthcare environments (55).

### 4.1 Limitation

This study offers valuable insights into the attitudes of psychiatrists in Tehran regarding COVID-19 stigma, as well as their perceptions of stigma associated with other medical conditions like AIDS and leprosy. However, several limitations should be acknowledged. The sample consisted of a limited number of psychiatrists (131), which may not fully represent the broader psychiatric community in Tehran or across Iran as a whole. A larger and more diverse sample could provide a more comprehensive understanding of stigma attitudes in this regard. Additionally, the study utilized a cross-sectional design by capturing attitudes at a single point in time. This method does not permit an analysis of changes in stigma perceptions over time or the influence of evolving public health policies and pandemic dynamics. Although psychiatrists are uniquely positioned to understand stigma, their attitudes may differ substantially from those of other HCWs or the general population. Future research could benefit from comparisons of stigma perceptions across various medical specialties and community groups. The study relied on self-reported survey responses, which are prone to biases such as social desirability bias; thus, the participants may have underreported or overreported their attitudes toward stigma based on perceived expectations or personal beliefs. While the study identified the persistence and overlap of stigma across different diseases, it did not investigate the specific cultural, societal, or institutional factors that drive these attitudes. Qualitative approaches could yield deeper insights into these underlying mechanisms. Furthermore, as the study was conducted in Tehran, its findings may not be generalizable to psychiatrists or HCWs in other regions of Iran or other parts of the world, where cultural and healthcare system differences may significantly impact stigma perceptions.

### 4.2 Suggestions for future studies

Based on the findings and limitations of the study, several directions for future research are proposed to enhance the understanding of stigma dynamics in healthcare settings and beyond. Future research should adopt longitudinal designs to investigate how attitudes toward COVID-19 stigma evolve, particularly as the pandemic progresses or recedes. This approach can reveal trends in the persistence or reduction of stigma, and also the effectiveness of interventions. Expanding the scope to include a broader range of healthcare professionals, including nurses, general practitioners, and frontline HCWs, as well as the general population can provide a more comprehensive perspective on stigma perceptions and their variation across different demographic and occupational groups. Incorporating qualitative components like interviews or focus groups can help uncover the cultural, societal, and institutional factors that influence stigma attitudes. This deeper exploration will complement quantitative findings and offer practical insights for addressing stigma. Conducting similar studies in diverse districts of Iran or in other countries will help identify cultural and systemic differences in stigma perceptions. Comparative studies could further highlight universal versus context-specific drivers of stigma. Finally, investigating how stigma impacts the mental health, job performance, and interpersonal relationships of HCWs can provide a more comprehensive understanding of its consequences and enlighten the development of targeted support systems.

## 5 Conclusion

The present study offers valuable insights into the attitudes of psychiatrists in Tehran regarding the stigma associated with COVID-19 and other infectious diseases such as AIDS and leprosy. The findings revealed a variety of attitudes, with the majority of psychiatrists acknowledging the persistence of stigma even as the pandemic evolved. Crucially, the research highlights the interconnected dynamics of stigma across different diseases, and emphasizes common societal factors like fear, misinformation, and cultural biases. Moreover, perceptions of stigma were not significantly influenced by demographic factors, including age, gender, marital status, or years of work experience. This suggests that stigma is a pervasive issue among psychiatrists, and so underscores the necessity for comprehensive educational interventions instead of targeted approaches. By exploring psychiatrists’ attitudes, the study puts an emphasis their vital role in combating stigma and its repercussions. As mental health professionals, psychiatrists are uniquely equipped to lead initiatives aimed at reducing stigma through public education, advocacy, and therapeutic interventions. Overall, the research findings suggest that recognition of stigma is crucial for cultivating a more inclusive healthcare system, enhancing mental health outcomes, and alleviating the societal consequences of pandemics.

## Data Availability

The raw data supporting the conclusions of this article will be made available by the authors, without undue reservation.
